# P-3. Investigating the Surge of Group C and G Streptococcal Bacteremia in a Rhode Island Community Hospital

**DOI:** 10.1093/ofid/ofaf695.234

**Published:** 2026-01-11

**Authors:** Angelica M Chan, Anais ovalle, Hadeel Zainah

**Affiliations:** Brown University/Kent Hospital, Providence, RI; Care New England/Brown University, West Lebanon, New Hampshire; Kent County-Memorial Hospital, Warwick, Rhode Island

## Abstract

**Background:**

*Streptococcus dysgalactiae* is a gram positive, beta-hemolytic bacteria of Lancefield’s groups C and G. Once considered non-pathogenic to humans, *S. dysgalactiae* is now recognized as a cause of diverse infections, from mild pharyngitis to life-threatening soft-tissue necrosis and bacteremia. Increasing incidence of invasive Group C/G streptococcal disease has been reported in western Norway and Finland, yet U.S. surveillance data remain limited.

**Figure:**
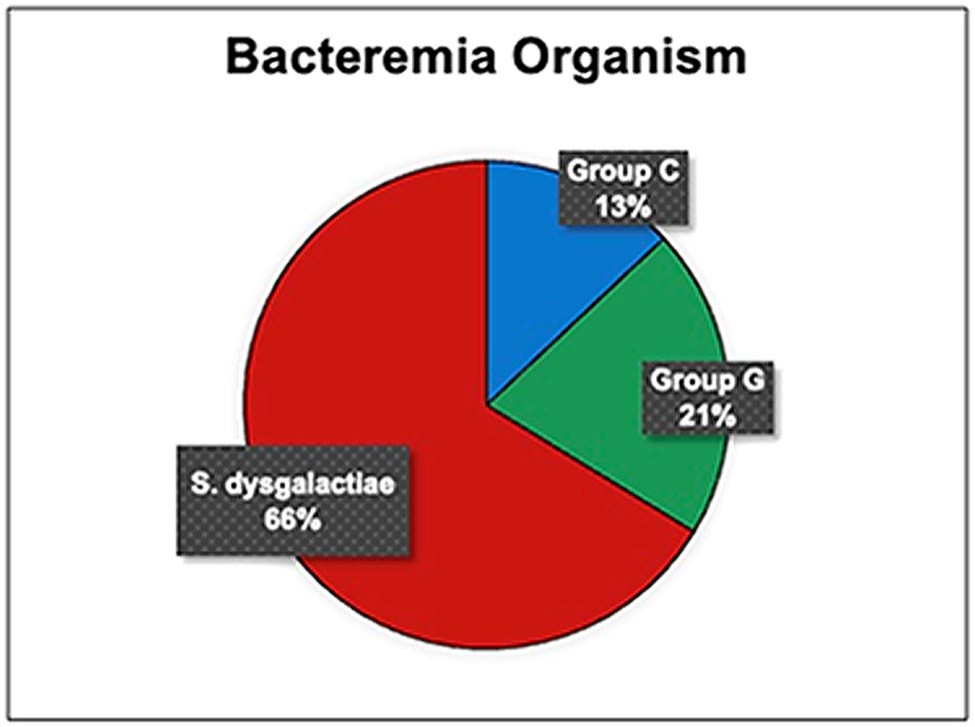

**Figure:**
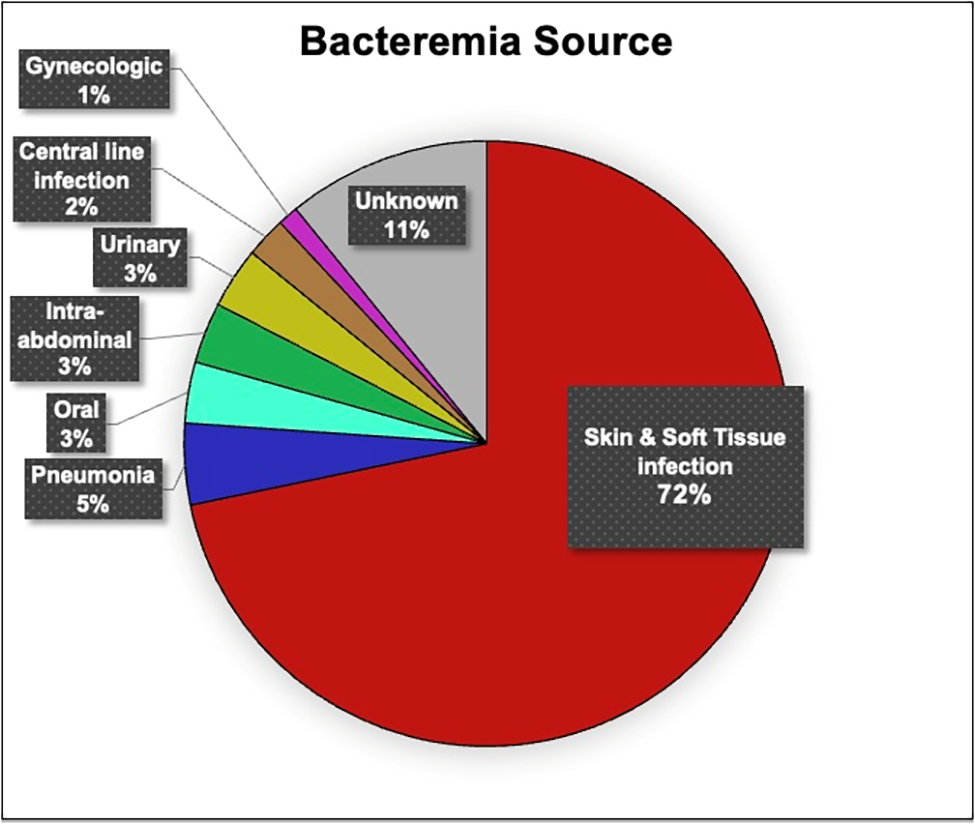

**Methods:**

With Institutional Review Board approval, we conducted a retrospective chart review to assess the burden of Group C/G/*S. dysgalactiae* bacteremia in a 359-bed acute care hospital in Rhode Island, USA. This study included 92 patients admitted between August 2015 and May 2024 with positive blood cultures for Group C or G streptococci or *S. dysgalactiae* subspecies. We collected and analyzed demographic (age, sex, comorbidities, race), clinical (bacteremia duration, co-infections, antimicrobial resistance), and outcome variables (length of stay, survival at discharge and at 6 moths). Patients were stratified into two cohorts: pre-pandemic (2015-2019) and pandemic/post-pandemic (2020-2024) to assess potential trends related to the COVID 19 era.

**Figure:**
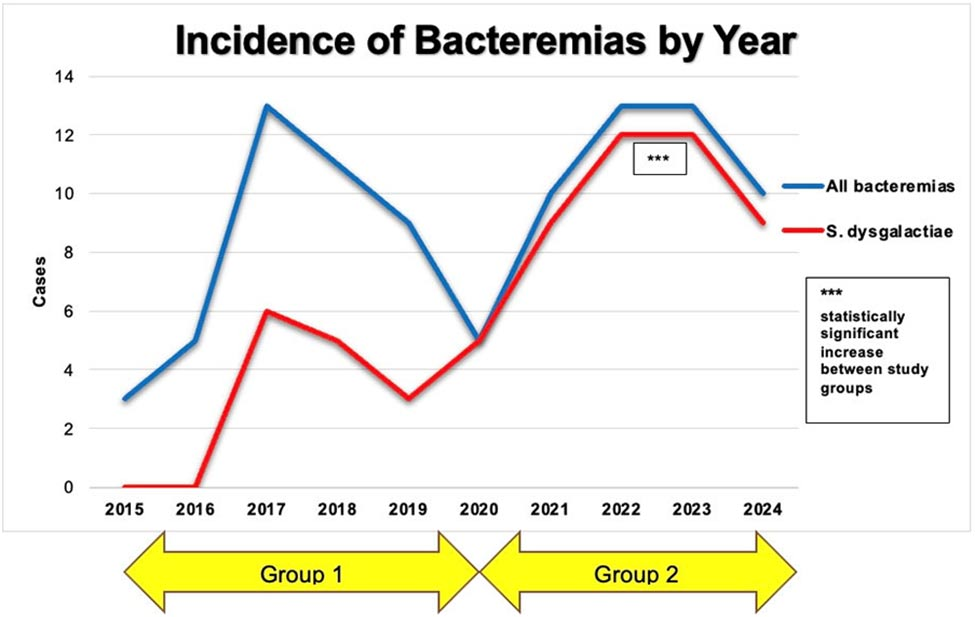

**Figure:**
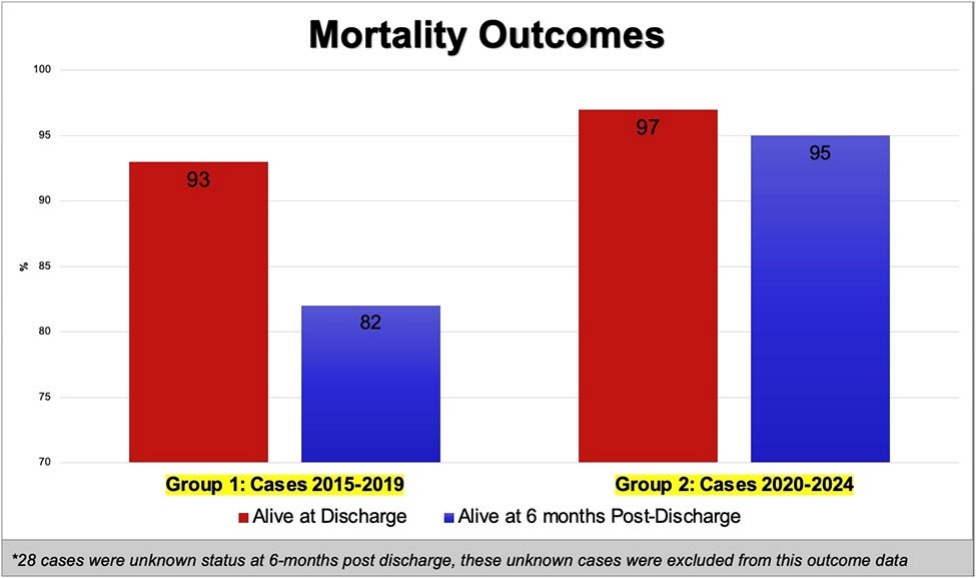

**Results:**

Most common co-morbidities were hypertension, diabetes mellitus, congestive heart failure and chronic kidney disease. Active malignancy at time of admission (7%) and immune suppression with known chemotherapy or immunomodulator (8%) was also noted. The primary bacteremia source was skin and soft-tissue infection in (72%). A statistically significant increase in Group C/G/*S. dysgalactiae* bacteremia was observed in 2020-2024 with *S. dysgalactiae* being the most isolated (p=0.0001). However, demographics, clinical variables, and study outcomes were similar between the two groups.

**Conclusion:**

*S. dysgalactiae* is a quickly emerging bacteremia causing pathogen. The significant post 2020 increase suggests a potential association with pandemic related factors. Further investigation is needed on how the COVID-19 pandemic may have created opportune environment for these invasive streptococcal infections.

**Disclosures:**

All Authors: No reported disclosures

